# Preoperative risk stratification for early recurrence of HBV-related hepatocellular carcinoma after deceased donor liver transplantation: a five-eight model development and validation

**DOI:** 10.1186/s12885-019-6343-4

**Published:** 2019-11-21

**Authors:** Abdulahad Abdulrab Mohammed Al-Ameri, Xuyong Wei, Lidan Lin, Zhou Shao, Haijun Guo, Haiyang Xie, Lin Zhou, Shusen Zheng, Xiao Xu

**Affiliations:** 1 Department of Hepatobiliary and Pancreatic Surgery, First Affiliated Hospital, Zhejiang University School of Medicine; Institution of Organ Transplantation, Zhejiang University, Hangzhou, 310003 China; 2 NHFPC Key Laboratory of Combined Multi-organ Transplantation, Hangzhou, 310003 Zhejiang Province China; 3 China Liver Transplant Registry, Hangzhou, China

**Keywords:** Liver transplantation, Hepatoma, Milan criteria, Hangzhou criteria,prognosis,relapse

## Abstract

**Background:**

Early recurrence of hepatocellular carcinoma (HCC) after liver transplantation (LT) is associated with poor surgical outcomes. This study aims to construct a preoperative model to predict individual risk of post-LT HCC recurrence.

**Methods:**

Data of 748 adult patients who underwent deceased donor LT for HCC between January 2015, and February 2019 were collected retrospectively from the China Liver Transplant Registry database and randomly divided into training (*n* = 486) and validation(*n* = 262) cohorts. A multivariate analysis was performed and the five-eight model was developed.

**Results:**

A total of 748 patients were included in the study; of them, 96% had hepatitis B virus (HBV) and 84% had cirrhosis. Pre-LT serum alpha-fetoprotein (AFP), tumor number and largest tumor diameter were incorporated to construct the 5–8 model which can stratify patients accurately according to their risk of recurrence into three prognostic subgroups; low-(0–5 points), medium-(6–8 points) and high-risk (> 8 points) with 2-year post-LT recurrence rate of (5,20 and 51%,*p* <  0.001) respectively. The 5–8 model was better than Milan, Hangzhou, and AFP-model for prediction of HCC early recurrence. These findings were confirmed by the results of the validation cohort.

**Conclusions:**

The 5–8 model is a simple validated and accurate tool for preoperative stratification of early recurrence of HCC after LT.

## Background

Globally, hepatocellular carcinoma (HCC) is the sixth most common malignancy and the third leading cause of cancer-related deaths [1]. In the Pacific region, China accounted for 84.6% of HCC incidence and 86.3% of HCC mortality with Hepatitis B virus (HBV) infection as the most common cause [2].

Although liver transplantation (LT) is an excellent therapeutic choice for HCC as the patients who received LT have the highest chance of cure among all other therapies, the organs shortage is still a main challenge [3]. For this, several selection criteria of HCC candidates for LT were proposed, of them, Milan and Hangzhou criteria are the most recommended tools by the Chinese Society of Organ Transplantation [4, 5]. The high rates of HCC recurrence after LT which have been reported to be 8–30% [3, 6] remains an important cause of death for HCC patients.

Alpha-fetoprotein (AFP) is a potential biomarker for early diagnosis and prediction of HCC recurrence. High level of preoperative AFP, which can be seen in approximately 60% of the HCC patients, is a risk factor for HCC recurrence and can be used to define at-risk HCC patients [7, 8]. In addition to AFP, there are well recognized preoperative risk factors which reflect the biological behavior of HCC and closely associated with post-LT HCC recurrence including vitamin K absence-II and neutrophil-to-lymphocyte ratio [9, 10]. The predominant morphological factors that show correlation with higher rates of HCC recurrence after LT include; tumor number and size [11, 12].

These risk factors were employed in a collective fashion to establish different predictive models for HCC recurrence, however unavailability of an effective, validated and reliable model to stratify patients preoperatively of HCC recurrence makes a unified practice across different countries out of reach. So, careful preoperative risk stratification of HCC recurrence is not only crucial for better management, but also very helpful to define a risk-based prioritizing strategy for selection of HCC candidates for LT.

In this multicenter study, we established a preoperative predictive model for early recurrence of HCC after LT. This model could be used as an adjuvant tool beside the conventional selection criteria to predict postoperative prognosis at a personal level more accurately.

## Methods

The design of this study followed the Transparent Reporting of a multivariable prediction model for Individual Prognosis or Diagnosis (TRIPOD) Statement [13]. This study has been approved by the Scientific Committee of the China Liver Transplant Registry (http://www.cltr.org) which is in accordance with ethical guidelines of Helsinki Declaration 1975, as revised in 2013. Written informed consent was obtained. Data of 1512 consecutive patients who underwent LT were retrospectively recalled from the prospectively maintained database of (CLTR) from 2015 January to 2019 February. Inclusion criteria were [1] adult patients with age ≥ 18 [2] pre-operative radiologically diagnosed HCC depending on guidelines of the current guidelines of American Association for the Study of Liver Diseases (AASLD) [14] [3] no history of previous LT or combined hepatorenal transplantation [4] patients who survived at least 3 months after the date of surgery [5] no incidental HCC [6] all the clinical and laboratory data required for the analysis are available. After applying the inclusion criteria, 748 patients were involved in the final analysis and divided randomly into training (*n* = 486) and validation (*n* = 262) cohorts. Data collection were performed by independent researchers blinded to statistical analysis. The collected clinicopathological variables included; age, gender, diabetes and hypertension, body mass index (BMI), presence of hepatitis B virus (HBV) infection, cirrhosis, Model for End-stage Liver Disease (MELD), Child score, neoadjuvant therapy (i.e. transarterial chemoembolization (TACE), radiofrequency ablation (RFA) and hepatectomy), donor type, donor death cause. The Pre-LT characteristics of HCC were obtained from radiological assessment (mainly CT, MRI), including the total tumor diameter, largest tumor diameter, number of nodules and the last pre-LT measurements of AFP. Post-LT features of HCC were obtained from the pathology reports including lymphovascular invasion and tumor differentiation according to the modified Edmondson score [15]. Data of survival and recurrence, including death cause, last follow-up dates, recurrence and death dates. Milan, Hangzhou criteria and AFP model were calculated [4, 5, 16]. The last censoring date of this study was 21st February 2019.

### Outcome and definitions

The primary outcome of this study is 2-year recurrence rate of HCC after LT. The recurrence was considered as ‘early’ if the time from LT to recurrence was ≤2 years [17]. Time to recurrence was calculated from the date of LT surgery to the date of recurrence diagnosis or last follow-up. The corresponding overall survival (OS) was also calculated from the date of LT surgery to date of death or last follow-up.

The selection criteria for LT included in this study are Milan and Hangzhou criteria. The former required the absence of distance metastasis and macrovascular invasion and included patients with a single nodule ≤5 cm or ≤ 3 nodules (each nodule ≤3 cm) [4], while the latter required the absence of macrovascular invasion and included patients with (a) total tumor size≤8 cm, (b) total tumor diameter > 8 cm, well moderate tumor differentiation and preoperative AFP level ≤ 400 ng/mL, concurrently [5].

AFP model is a binary tool incorporated largest tumor size, number of nodules and pre-LT AFP (at 100 and 1000 ng/mL) with a cut-off value of two points to discriminate patients within and outside the AFP model patients [16].

The death was defined as HCC- related death if there is an evidence of HCC recurrence post-LT or documented metastasis and/or vascular invasion otherwise it was considered as HCC-unrelated death. The last censoring date or the date of events (recurrence and death) were considered after following up all patients.

### Postoperative management and follow up

Generally, postoperative immunosuppressants consisted of calcineurin inhibitors and steroids. Steroids were withdrawn within 3 months. Follow up of the patients were done every 3–6 months during the first 2 years post-LT. During follow up time, in addition to AFP measurement, abdominal computed tomography (CT) scan and magnetic resonance imaging (MRI) were performed according to their indications.

### Statistical analysis

Statistical analysis was performed using Stata MP 14. Categorical data were reported as values and percentages and compared using Fisher’s exact test or Chi-Square test. Continuous data were reported as mean ± SD or median (interquartile range [IQR]) and compared with Student’s T-test or rank sum test according to their distribution respectively. Recurrence and survival probabilities were estimated by Kaplan-Meier (KM) methods and compared using the log-rank test (Mantel-Cox). Univariable and multivariable Cox regression analyses for factors affecting post-LT HCC recurrence were performed by Cox proportional hazards regression models. Variables after a univariable analysis with a *P*-value < 0.05 were included in the multivariable analysis, and the final model was constructed by stepwise backward selection (Wald test). Notebaly,the potential cut-off values were estimated in accordance with previous studies in the literature and using the Akaike information criterion (AIC), the cut-off points with the lowest AIC values were selected to be included in the final model. Proportional-hazards assumption was assessed by the Schoenfeld test and by visual assessment of log-log survival curves. The training and validation cohorts were compared. The discriminatory performance of 5–8 Model, Milan, Hangzhou, and AFP model was calculated and compared using Harrell’s C and Somers’ D statistics [18] and also was assessed visually via KM curves. Moreover, the competing risk analysis was also performed to evaluate the cumulative incidence of HCC related and unrelated deaths [19]. A two-tailed *p*-value of < 0.05 indicates a statistically significance difference.

## Results

**Fig. 1:**
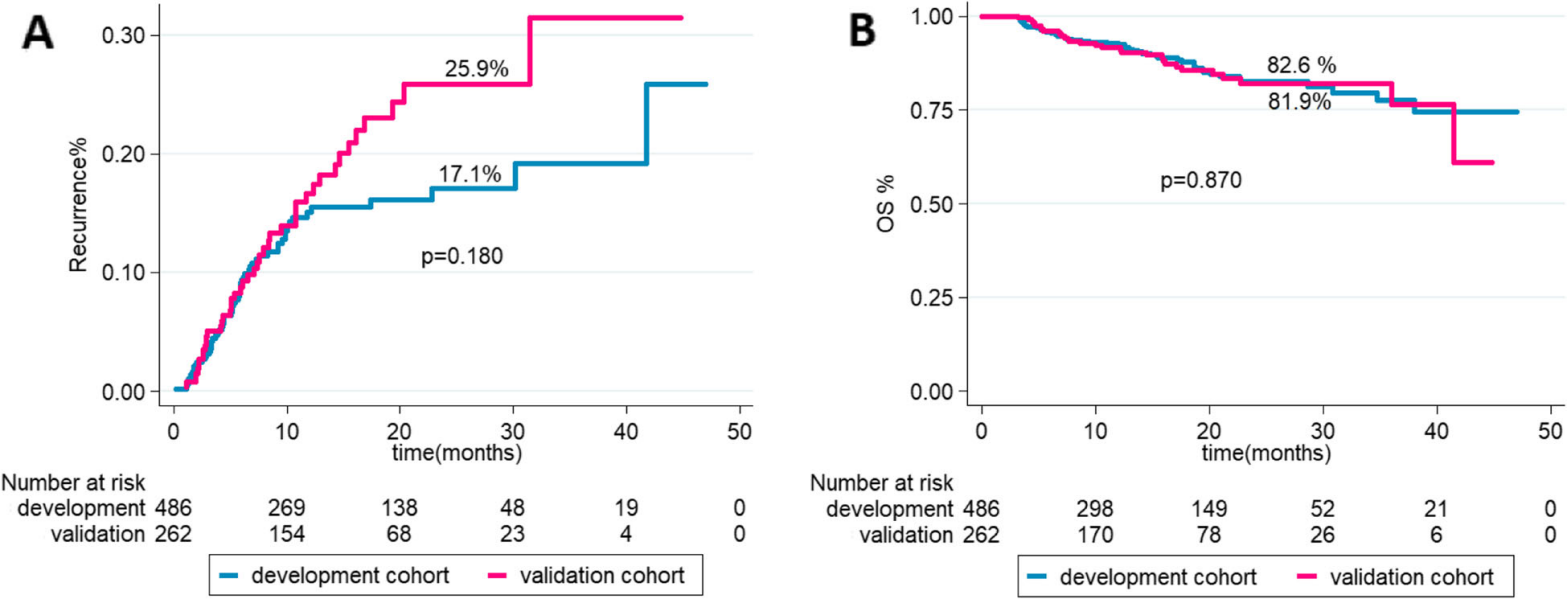
The 2-year recurrence and overall survival rates in the training (**a**) and validation(**b**) cohorts

A total of 748 patients were included in the study with a mean age of 51.6 ± 8.6 and 89.6% were male. HBV infection was the most common cause (96%) and cirrhosis was found in 84%. The 486 patients of the training cohort had similar characteristics to the 262 patients of validation cohort without any significant differences as summarized in (Table 1). For the training and validation cohorts, the median post-LT follow-up was 338 days, (IQR: 205–673 days) and 416.5 days, (IQR:205–672 days), respectively. The 2-year OS was 82.6% (95%CI:0.77–0.87) vs 81.9% (95% CI: 0.74–0.87), *p* = 0.870(Fig. 1a). Recurrence of HCC was observed in 12.8% (62 of 486) vs 16.8% (44 of 262) at a median of 11.3 months (IQR: 5.9–21.2, months) vs 12.0 months IQR:6.2–20.5 months) after LT. The 2-year overall HCC recurrence was 17.1% (95% CI:0.13–0.22) vs 25.9% (95% CI: 0.19–0.36), *p* = 0.180 (Fig. 1b).

**Table 1:** Baseline characteristics of training and validation cohorts

Variable	Training cohort (n = 486)	Validation cohort(n = 262)	*P*-value
Age (years)^a^	51.6 ± 8.6	51.5 ± 8.7	0.890
Gender (Male/Female) ^b^	439 (90.3)/47 (9.7)	231 (88.1)/31 (11.8)	0.356
Diabetes (Yes/No) ^b^	62 (12.8)/424 (87.2)	40 (15.3)/222 (84.7)	0.340
Hypertension (Yes/No) ^b^	48 (9.9)/438 (90.1)	33 (12.6)/229 (87.4)	0.254
BMI^a^	24.4 ± 12.9	23.3 ± 3.8	0.162
Cause (HBV/Non-HBV) ^b^	462 (95.1)/24 (4.9)	256 (97.7)/6 (2.3)	0.078
Cirrhosis (Yes/No) ^b^	411 (84.6)/75 (15.4)	217 (82.8)/45 (17.2)	0.535
Pre-LT AFP (ng/mL) ^b^			
≤ 10	164 (33.7)	94 (35.9)	0.621
10–200	167 (34.4)	95 (36.3)	
200–1000	82 (16.9)	35 (13.4)	
> 1000	73 (15.0)	38 (14.5)	
Total tumor diameter (cm)^b^			
≤ 5	283 (58.2)	148 (56.5)	0.880
5.1–8	104 (21.4)	57 (21.8)	
> 8	99 (20.4)	57 (21.8)	
Largest tumor diameter (cm)^b^			
≤ 4	309 (63.6)	162 (61.8)	0.296
4–6	83 (17.1)	56 (21.4)	
6.1–8	42 (8.6)	15 (5.7)	
> 8	52 (10.7)	29 (11.1)	
Tumor number (single/multiple) ^b^	296 (60.9)/190 (39.1)	151 (57.6)/111 (42.3)	0.384
MELD^c^	12 [8–21], (6–51)	11 [9–19], (6–44)	0.503
Child score^c^	7 [5–9], (5–14)	7 [5–9], (5–14)	0.473
Neoadjuvant therapy^b^			
TACE (yes/no)	195 (40.1)/291 (59.9)	113 (43.1)/149 (56.9)	0.425
RFA (yes/no)	85 (17.5)/401 (82.5)	49 (18.7)/213 (81.3)	0.680
Hepatectomy (yes/no)	83 (17.1)/403 (82.9)	41 (15.7)/221 (84.4)	0.616
Donor type ^b^ DBD/DCD/DBCD	142 (29.2)/174 (35.8)/170 (35.0)	70 (26.7)/103 (39.3)/89 (34.0)	0.609
Donor death cause ^b^			
Trauma	233 (47.9)	138 (52.7)	0.404
CVA	190 (39.0)	94 (35.9)	
Tumor	29 (6.0)	10 (3.8)	
Anoxia	17 (3.5)	13 (4.9)	
Others	17 (3.5)	7 (2.7)	
Differentiation ^b^			
well	72 (14.8)	42 (16.0)	0.884
moderate	338 (69.6)	178 (67.9)	
poor	76 (15.6)	42 (16.0)	
Vascular invasion (yes/no) ^b^	113 (23.3)/373 (76.8)	74 (28.2)/188 (71.8)	0.132
Milan (in/out) ^b^	259 (53.3)/227 (46.7)	125 (47.7)/137 (52.3)	0.145
Hangzhou (in/out) ^b^	390 (80.3)/96 (19.8)	197 (75.2)/65 (24.8)	0.108
AFP model (in/out) ^b^	301 (61.9)/185 (38.1)	167 (63.7)/95 (36.3)	0.626
Post-LT mortality (Died/alive) ^b^	55 (11.3)/431 (88.7)	31 (11.8)/231 (88.2)	0.833
Post-LT recurrence (yes/no) ^b^	62 (12.8)/424 (87.2)	44 (16.8)/218 (83.2)	0.131
Time to recurrence (months)^c^	11.3 [5.9–21.2], (0.2–47.0)	12.0 [6.2–20.5], (1.1–44.8)	0.863
Follow-up (days)^c^	388 [205–673], (92–1428)	416.5 [205–672], (92–1363)	0.802

Note: *BMI* Body mass index, *HBV* Hepatitis B virus infection, *AFP* Alpha-fetoprotein, *MELD* Model for End-Stage Liver Disease, *LT* Liver transplantation, *TACE* Transarterial chemoembolization, *RFA* Radiofrequency ablation, *DBD* Donation after brain death, *DCD* Donation after circulatory death, *DBCD* Donation after brain death followed by circulatory death, *CVA* Cerebrovascular accident, ^a^Mean ± SD, ^b^ number (percentage), ^c^ (median, [IQR, interquartile range]),(range)

### Factors affecting post-LT HCC recurrence

Factors of post-LT HCC recurrence in the training cohort were identified by univariable Cox regression analysis (Table 2). The preoperative factors that associated with post-LT HCC recurrence included pre-LT TACE, pre-LT AFP, total tumor diameter (cm), the largest tumor diameter (cm) and the number of nodules at all tested cut-off values. The postoperative factors included; vascular invasion, poorly differentiated tumor grade. Other factors including recipient age, gender, MELD score, number of HCC nodules, use of other neoadjuvant therapies (i.e. hepatectomy, RFA) did not associate with post-LT HCC recurrence. We emphasize that the tumor diameters and the number of nodules were obtained from the last imaging before transplantation by which Milan and Hangzhou criteria were calculated. On multivariable analysis, only pre-LT AFP, largest tumor size and tumor number were the preoperative predictors found to be associated with increasing the risk of post-LT HCC recurrence. While the postoperative predictors were the only presence of vascular invasion and poorly differentiated tumor grade.

**Table 2:** Univariable cox analysis of risk factors for early recurrence of HCC

Variable	2-year recurrence rate	HR	SE	z	*P* > z	95% CI
Age (52/≤52 years)	17.6/16.4	0.98	0.25	0.07	0.942	0.59–1.63
Gender (male/female)	17.9/9.2	1.51	0.78	0.80	0.426	0.55–4.16
Diabetes (yes/no)	19.4/17.1	0.79	0.34	0.55	0.585	0.34–1.84
Hypertension (yes/no)	24.7/16.3	1.54	0.59	1.14	0.253	0.73–3.25
BMI (25/≤25)	18.5/16.4	0.99	0.28	0.05	0.959	0.561.73
CTP class(A/B/C)	19.5/14.9/15.3	0.94	0.16	0.38	0.705	0.67–1.31
MELD (12/≤12)	13.7/20.2	0.81	0.21	0.81	0.416	0.49–1.35
Cirrhosis (yes/no)	17.414.2	1.24	0.47	0.57	0.566	0.59–2.62
Pre-LT AFP (ng/mL) (Reference, ≤10)						
10–200	19.5	3.57	1.65	2.75	0.006	1.44–8.83
201–1000	21.8	4.65	2.29	3.11	0.002	1.77–12.23
> 1000	39.2	9.03	4.20	4.72	< 0.001	3.62–22.49
Total tumor diameter (cm) (Reference, ≤5)						
5–8	21.9	3.20	1.19	3.13	0.002	1.55–6.64
> 8	52.6	8.89	2.88	6.75	< 0.001	4.7116.76
Largest tumor diameter (cm) (Reference, ≤4)						
4–6	21.8	2.37	0.90	2.28	0.023	1.13–4.98
6–8	28.2	3.67	1.55	3.08	0.002	1.61–8.39
> 8	74.8	10.49	3.31	7.45	< 0.001	5.65–19.46
Nodules number (single/multiple)	12.3/24.6	1.883142	.4874233	2.45	0.014	1.13–3.13
Pre-LT TACE (yes/no)	23.3/13.0	2.03	0.53	2.73	0.006	1.22–3.39
Pre-LT RFA (yes/no)	13.6/18.0	0.79	0.29	0.65	0.519	0.39–1.61
Pre-LT hepatectomy (yes/no)	25.0/15.5	1.59	0.49	1.52	0.128	0.88–2.90
Vascular invasion (yes/no)	43.0/9.8	4.31	1.12	5.62	< 0.001	2.59–7.17
Differentiation (Reference, well)						
moderate	14.9	1.52	0.67	0.95	0.344	0.64–3.59
poor	26.1	2.81	1.35	2.16	0.031	1.10–7.19
Milan criteria (out/in)	33.8/4.8	7.68	2.78	5.64	< 0.001	3.78–15.62
Hangzhou criteria (out/in)	57.5/9.1	7.24	1.91	7.52	< 0.001	4.32–12.13
AFP model (out/in)	35.0/7.9	5.80	1.73	5.89	< 0.001	3.23–10.41

Note: *BMI* Body mass index, *CTP* Child-Turcotte-Pugh, *AFP* Alpha-fetoprotein, *MELD* Model for End-Stage Liver Disease, *LT* Liver transplantation, *TACE* Transarterial chemoembolization, *RFA* Radiofrequency ablation

### Development of the 5–8 score

Preoperative factors with *p*-value < 0.05 on univariable analysis were then used in the multivariable model. A Cox regression analysis was then performed with backward selection to conduct a multivariable analysis of clinicopathologic factors that associated significantly with post-LT HCC recurrence. Preoperative independent factors of post-LT HCC recurrence were utilized to construct the 5–8 score including (1) pre-LT AFP (at the following cut-off values: 10–200, 201–1000, and > 1000 ng/mL), (2) the largest diameter of tumor (at the following cut-off values: 4–6,6.1–8 and > 8), and (3) number of nodules (single vs multiple). It is important to note that the model with the lowest (AIC), was chosen as the final model for the risk score. The multivariable HR of these factors derived from the Cox regression model were rounded to the nearest integer, then used to calculate the simplified 5–8 score. To calculate the score for each patient, the individual points for each of the three variables can be added together giving a minimum point of 0 and a maximum point of 24(Table 3).

**Table 3:** Multivariate Cox analysis of risk factors for early recurrence of HCC

Variable	HR	SE	z	P > z	95% CI	β Coefficient	Points
Pre-LT AFP (ng/mL)							
≤ 10	1	–	–	–	–	–	0
10–200	3.12	1.51	2.29	0.022	1.18–8.25	1.29	3
200–1000	3.62	1.69	2.77	0.006	1.46–9.05	1.14	4
> 1000	6.22	2.92	3.89	< 0.001	2.48–15.62	1.83	6
Largest tumor diameter (cm)							
≤ 4	1	–	–	–	–	–	0
4–6	2.17	0.83	2.03	0.043	1.01–4.57	0.77	2
6–8	3.09	1.32	2.64	0.008	1.34–7.16	1.13	3
> 8	12.82	4.43	7.39	< 0.001	6.52–25.24	2.55	13
Nodules number							
Single	1	–	–	–	–	–	0
Multiple	2.54	0.71	3.36	0.001	1.48–4.38	0.93	3

0–5: low risk, 6–8 medium risk, > 8 high risk

### Prediction of HCC recurrence risk by the 5–8 score

**Fig. 2:**
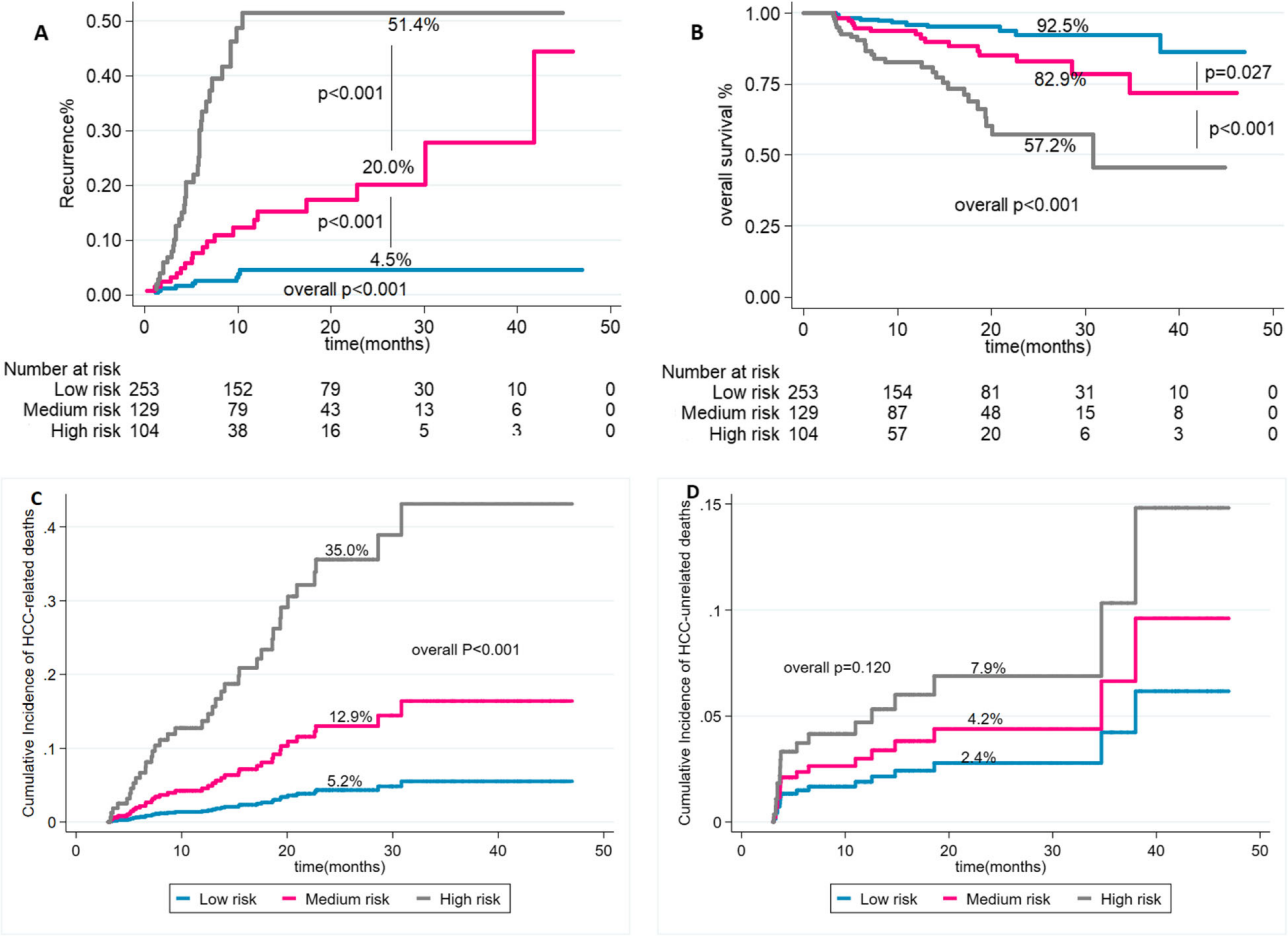
In the training cohort and according to the 5–8 model, the 2-year recurrence rates (**a**) and overall survival rates(**b**). The cumulative incidence of HCC-related deaths (**c**) and HCC-unrelated deaths(**d**) as assessed by the competing risk analysis

Based on the 5–8 score, patients were then accurately stratified according to their risk of recurrence into three categories; the low-risk group had a score of 0 to 5, the medium-risk group had a score of 6–8, the high-risk group had a score of > 8(Table 3). The most common group being low risk group [*n* = 253(52.1%)] then medium-risk group [*n* = 129(26.5%)] and high-risk group (*n* = 104(21.4%)]. The risk of 2-year HCC recurrence was increased significantly from low to high-risk group as shown by KM curves and log-rank test. The 2-year HCC recurrence rate was 4.5% (95% CI:0.02–0.09), 20.0% (95% CI:0.12–0.34) and 51.4% (95% CI:0.36–0.73) in the low, medium and high-risk group respectively (overall log-rank *p* <  0.001))(Fig. 2a). The corresponding 2- year OS was 92.5% (95% CI: 0.86–0.96), 82.9%(95% CI:0.72–0.90) and 57.2% (95% CI: 0.42–0.70), respectively (overall log-rank *p* < 0.001) (Fig. 2b). According to the 5–8 score, patients before transplantation with AFP level of ≤10 ng/mL and single nodule with the largest tumor diameter of < 4 cm on radiological assessment would be categorized in the low-risk group, in contrast to patients who have pre-LT AFP of > 1000(ng/mL) and largest tumor diameter of > 8 cm, will be categorized in high-risk category. There was no deviation from the proportional hazard assumption according to the visual inspection of log-log survival curves and the Schoenfeld test (*p* = 0.708). Also, for prediction HCC recurrence in the training cohort, visual assessment of the KM curves showed good discrimination between the three 5–8 model prognostic subgroups. Moreover, the Harrell’s C and Somers’ D of 5-8score were 79%(95% CI:0.73–0.86) and 59%(95% CI:0.40–0.72) (Table 4). Based on the competing risk analysis, the 2-year cumulative incidence of mortality, while controlling for the risk of HCC-related death, was 5.2%(95% CI: 0.02–0.11), 12.9%(95% CI: 0.06–0.22) and 35.0% (95% CI: 0.22–0.49, overall *p* < 0.001) in patients with low, medium and high 5–8 score (Fig. 2c). Furthermore, 2- year cumulative incidence of HCC-unrelated death which not related to HCC recurrence were, 2.4%(95% CI: 0.01–0.05),4.2% (95% CI: 0.01–0.10) and 7.9%(95% CI: 0.03–0.16), (overall *p* = 0.120) in patients with low, medium and high 5-8score (Fig. 2d).

**Table 4:** Accuracy of the 5–8 model for predicting the risk of HCC early recurrence in the training and validation cohort compared with Milan, Hangzhou, and AFP model

	Training cohort		Validation cohort	
	Harrell’s C (95% CI)	Somer’s D (95% CI)	Harrell’s C (95% CI)	Somer’s D (95% CI)
5–8 model	0.79 (0.73–0.86)	0.59 (0.40–0.72)	0.74 (0.66–0.82)	0.49 (0.32–0.74)
Milan	0.72 (0.67–0.76)	0.43 (0.35–0.58)	0.65 (0.59–0.71)	0.30 (0.17–0.45)
Hangzhou	0.72 (0.65–0.78)	0.43 (0.31–0.61)	0.61 (0.54–0.69)	0.23 (0.07–0.40)
AFP model	0.72 (0.66–0.77)	0.43 (0.33–0.60)	0.68 (0.60–0.75)	0.35 (0.20–0.53)

### Comparison of the 5–8 model with Milan and Hangzhou criteria

**Fig. 3:**
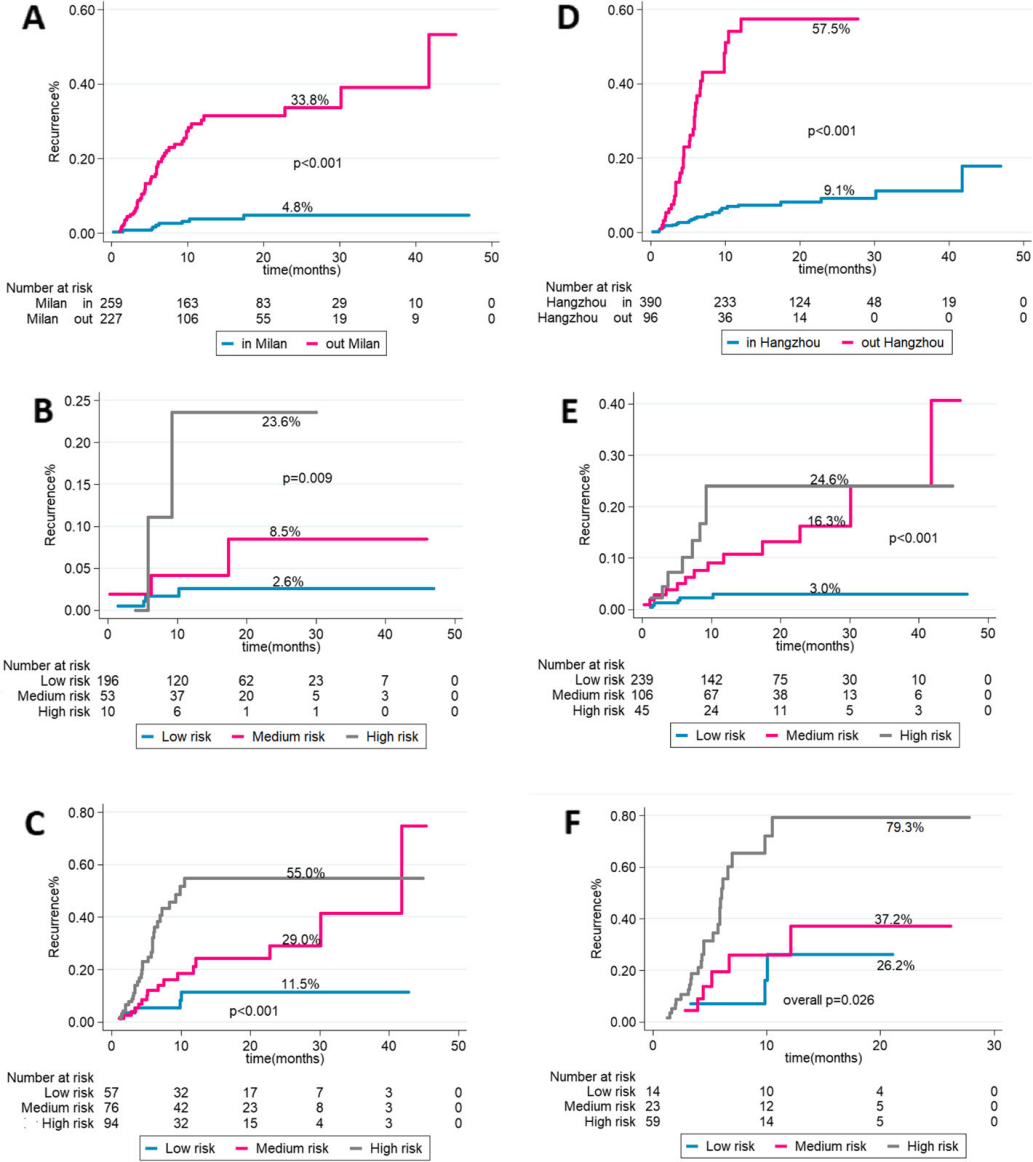
In the training cohort, 2-year recurrence rates according to Milan criteria (**a**), according to 5–8 model in patients fulfilling Milan criteria (**b**) and in patients exceeding Milan criteria (**c**). Two-year recurrence rates according to Hangzhou criteria (**d**), according to the 5–8 model in patients fulfilling Hangzhou criteria (**e**) and in patients exceeding Hangzhou criteria (**f**)

The 2-years recurrence rate for patients meeting and exceeding Milan criteria, was 4.8 and 33.8% respectively (*p* < 0.001) (Fig. 3a), while it was 9.1% vs 57.5% in patients meeting and exceeding Hangzhou criteria respectively, (*p* < 0.001) (Fig. 3d). We further analyzed the risk-stratified patients of 5–8 score for the patients who were fulfilling and exceeding either Milan or Hangzhou criteria. Among 259 patients who were fulfilling Milan criteria (53.2%), the risk of 2-year HCC recurrence according to 5–8 score was 2.6% (95% CI: 0.01–0.07) in the low risk group, 8.5% (95% CI: 0.03–0.29) in medium- risk group and 23.6% (95%CI: 0.06–0.95) in the high-risk group (overall *p* = 0.009) (Fig. 3b). While for 227 patients who are exceeding Milan criteria (46.7%), the risk of 2-year HCC recurrence was 11.5% (95% CI: 0.05–0.28),29.0% (95% CI:0.16–0.51) and 55.0%(95% CI: 0.38–0.79), respectively (overall *p* < 0.001) (Fig. 3c). Likewise, for the 390 patients within Hangzhou criteria (80.3%), the risk of 2-year HCC recurrence according to 5–8 score was 3.0% (95% CI:0.01–0.07) in the low risk group, 16.3% (95% CI:0.09–0.31) in medium- risk group and 24.6% (95% CI: 0.12–0.49) in the high-risk group (overall *p* < 0.009) (Fig. 3e). While for 96 patients who are exceeding Milan criteria (19.8%), the risk of 2-year HCC recurrence was 26.2% (95% CI: 0.08–0.82), 37.2%(95% CI: 0.16–0.87) and 79.3% (95% CI:0.52–1.21), respectively (overall *p* < 0.001) (Fig. 3f). For prediction of HCC recurrence, Harrell’s C and Somers’ D of 5-8score were 79%(95% CI:0.73–0.86) and 59%(95% CI:0.40–0.72) in the training cohort compared with 72%(95% CI:0.67–0.76) and 43%(95% CI:0.35–0.58) for Milan criteria and 72%(95% CI:0.65–0.78) and 43%(95% CI:0.31–0.61) for Hangzhou criteria (Table 4).

### Comparison of the 5–8 model with AFP model

**Fig. 4:**
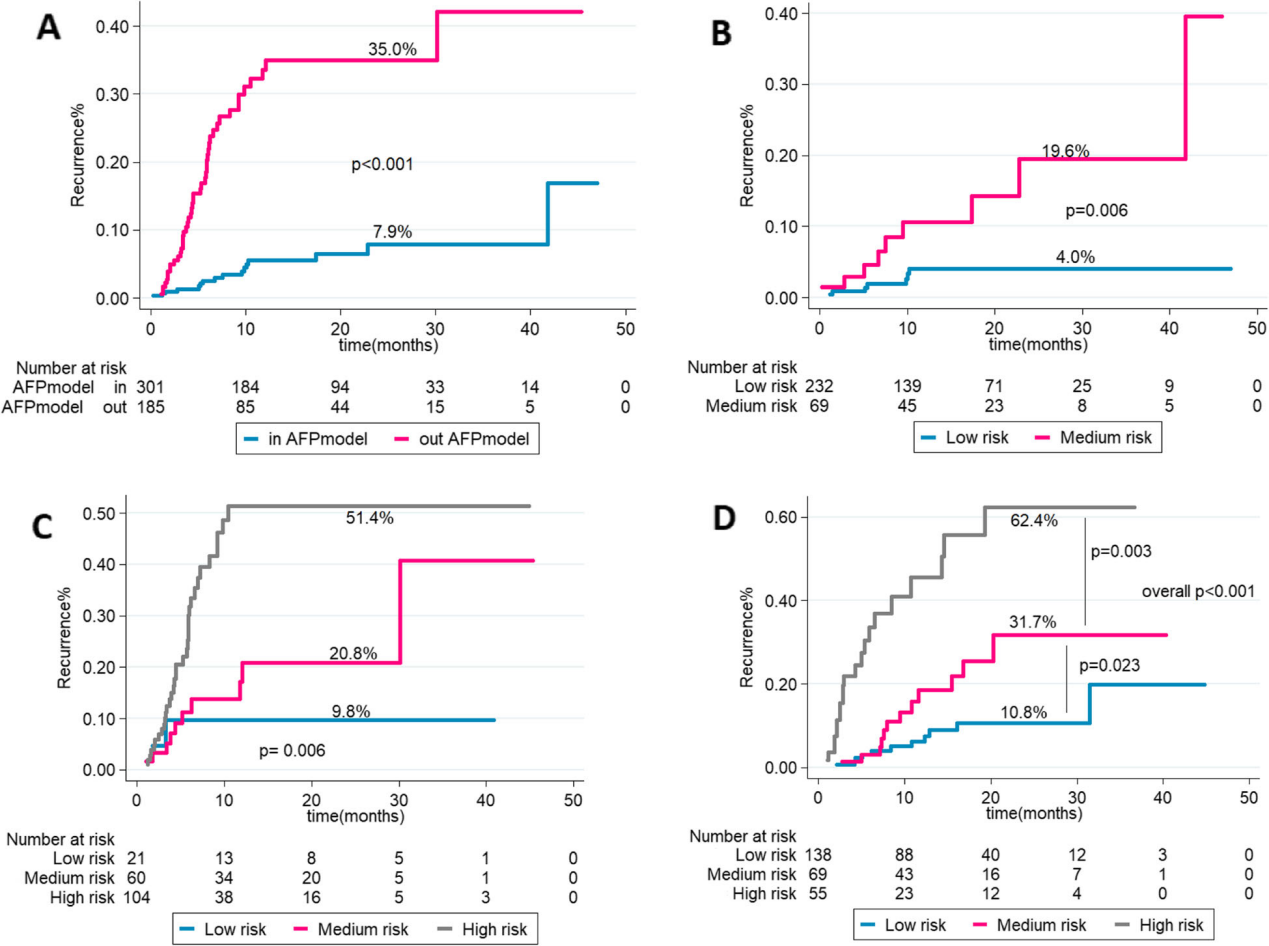
In the training cohort, the 2-year recurrence rates according to AFP model (**a**), according to the 5–8 model for patients within the AFP model (**b**) and in patients outside AFP model (**c**). In the validation cohort, the 2-year recurrence rates (**d**) according to the 5–8 model

For patients exceeding and fulfilling the AFP model, the 2-year rates of HCC recurrence were 35.0 and 7.9%, respectively (Fig. 4a). Further analysis for the risk-stratified patients of 5–8 score for the patients who were within and outside the AFP model showed that among 301 patients who were within AFP model (62%), the risk of 2-year HCC recurrence was 4.0% (95% CI: 0.02–0.09) in the low-risk group, 19.6% (95% CI: 0.09–0.43) in medium- risk group (*p* = 0.006) (Fig. 4b). While for 185 patients who are exceeding AFP model (38.1%), the risk of 2-year HCC recurrence was 9.8% (95% CI: 0.02–0.39),20.8% (95% CI: 0.11–0.41), and 51.4%(95% CI: 0.36–0.73), respectively (overall *p* = 0.006) (Fig. 4c).

### Validation of the 5–8 model

As mentioned above, there were no significant differences in baseline characteristics among the training and validation cohort. In the validation cohort, the median post-LT follow-up was 416.5 days [IQR:205–672]. The 5–8 model could also accurately stratify patients into low, medium and high-risk prognostic subgroups with 2-year HCC recurrence rates of 10.8% (95% CI: 0.06–0.21), 31.7% (95% CI: 0.17–0.59) and 62.4% (95% CI: 0.39–1.00) respectively, (overall log-rank *p* < 0.001) (Fig. 4d). The 5–8 score also achieved a good performance in post-LT recurrence risk prediction in the validation cohort according to the visual assessment of the resulting KM curves and the Harrell’s C and Somers’ D of 74%(95% CI:0.66–0.82) and 49%(95% CI:0.32–0.74) compared with 65%(95% CI:0.59–0.71) and 30%(95% CI: 0.17–0.45) for Milan criteria and 61%(95% CI:0.54–0.69), 23%(95% CI:0.07–0.40) for Hangzhou criteria (Table 4).

## Discussion

Recurrence of HCC after LT remains a major obstacle and associated with an unfavorable prognosis [20]. Several independent risk factors for post-LT HCC recurrence have been identified including number and size of tumors on preoperative imaging studies [11, 12, 21] and pre-LT serum levels of AFP at different cut-off points: 10,200,1000 ng/mL [22–25]. However, lack of agreement about accurate, reliable and robust validated tool especially for prediction of early recurrence of HCC in HBV predominant population, make appropriate risk stratification and doctor-patient communication challenging.

In the present study, 786 patients with HCC diagnosed by imaging who underwent deceased donor LT from centers distributed throughout the whole China were involved and the 5–8 score was developed and validated. Our predictive model is a simple and reliable tool that showed excellent stratification of HCC patients into three risk subgroups; low-, medium- and high-risk with predicted 2-year recurrence ranging from 5% in low-risk to 20.0% in the medium risk and 51% in the high-risk category. For the 253 patients (52.1%) in the low-risk group, the 2- year OS was 93%, significantly superior to the 83 and 57% of the medium- and high-risk groups.

Similar to our study, previous risk models [16, 26–28] have attempted to employ pre-LT AFP, largest tumor diameter and the number of nodules to predict the post-LT HCC recurrence, but the characteristics of their study population (western participants with predominantly HCV) was different. Our model is mainly based on the data of patients with HCC occurring in patients with HBV infection (96%) which is the most common cause of HCC in China. The high predictive accuracy of our preoperative model [Harrell’s C was 79%(95% CI: 0.73–0.86) and 74%(95% CI:0.66–0.82), respectively, in the training and validation cohorts] stems from the utilization of three preoperative factors with more accurate cut-off points that were strongly associated with HCC recurrence: pre-LT AFP(10–200, 201–1000, and > 1000 ng/mL), largest tumor diameter(4–6,6.1–8,> 8 cm), and the number of nodules (single vs multiple). Unlike previous studies, our model end-point was recurrence at 2-year which is the discriminative cut-off value for early and late recurrence of HCC, however, the precise cut-off may require genetic/genomic analyses recurrence. Early recurrence results from preexisting tumor cells while late recurrence or de novo tumor mainly arising due to new malignant clones [29–31]. Using the cut-off values in our study was a clinical decision based on the expert’s assessment and they achieved the lowest AIC values so better fit the model.

Furthermore, one of the main advantage of our score over previous mentioned models is its ability to discriminate three subgroups of HCC recurrence(i.e low,medium,high), while other models only classify HCC patients into two risk subgroups of HCC recurrence, high and low risk. The categorization defining only two subgroups at risk is not valuable, or at least less practical. For example, tumour recurrence risk divided into low (< 8%) and high (> 50%) will produces a big ‘grey zone’ through 8–50% of medium-risk individuals [32]. Our score solved this drawback by stratify the individuals at risk into three subgroups of risk of recurrence, low,medium and high. This can be translated clinically into an excellence of individuals selections for LT, a more reasonable organs allocation taking into account the donor offer, besides, and the opportunity to stratify risk for the development of upcoming adjuvant treatments [33].

One of the most interesting results comes when we compared our model with the conventional selection criteria by which our model can stratify patients within and outside the Milan and Hangzhou criteria. For instance, the subgroup of patients (25%) who were recognized as high risk of recurrence by Milan (out Milan), they carry a low risk according to our score with 2-year recurrence probability of 12%. While the subgroup of patients(4%) who were recognized as low risk of recurrence by Milan (in Milan), they carry a high risk according to our score with 2-year recurrence probability of 24%. Similarly, the subgroup of patients (12%) who were recognized as low risk of recurrence by Hangzhou (in Hangzhou), they carry a high risk according to our score with 2-year recurrence probability of 25%. The addition of our model to the conventional selection criteria may, therefore, allow us to capture accurately the patients with a higher risk of recurrence who were traditionally considered as the lowest risk group. External validation of this preoperative adjuvant model is mandatory to help in more accurate selection of HCC patients for LT and to avoid the high post-LT recurrence probabilities.

Moreover, one of the advantages of our model is when it was compared to the AFP model, about (11 and 32%) of patients recognized as high risk of recurrence by AFP model, but they had a low and medium risk according to our score with 2-year recurrence probability of 10, 21% respectively. Also, about 23% of the patients were defined as a low risk of recurrence by the AFP model, but a medium risk of recurrence was revealed by our score with 2-year recurrence probability of 20%. Another advantage of our model is the outcome of the competing risk analysis, the rates of HCC-unrelated death were similar (*p* = 0.120) and the rates of HCC-related death which mainly due to HCC recurrence were significantly different (*p* < 0.001), indicating that the 5–8 model is a powerful tool to pick up the HCC recurrence but not other causes of deaths and this explains the differences in survival rates according to the 5–8 model.

At this point, we need a further explanation for the clinical application of our model. The patients presented preoperatively with a single nodule, diameter of ≤4 cm and serum AFP of ≤10 ng/mL will fall in the low-risk group by getting the 5–8 score of 0 and this will effectively exclude the probability of post-LT HCC recurrence, so these patients can go directly for LT. However,The patients with largest nodule diameter of > 8 cm and serum AFP of > 1000 ng/mL will be categorized in the high-risk category (Table 3), so LT should be excluded or neoadjuvant therapy and close surveillance until the tumor biology and morphology could be brought down to a safer level. However, patients who belong to the medium HCC recurrence risk category (e.g. single nodule, diameter of 8 cm and serum AFP of 700 ng/mL), a careful selection for LT based on a personalized assessment and pre-LT downstaging therapy should be considered then early administration of mTOR inhibitor postoperatively are strongly recommended [34].

Our study has some limitations. First, its retrospective nature so we designed a prospective study to confirm the clinical utility of our model. Second, the characteristics of our study population (predominantly HBV infected male Chinese patients) so external validation in HCV predominant, non-Chinese populations is required to confirm the reliability of the 5–8 model. Third, the patients with vascular invasion were not excluded from our study. Fourth,some parameters, such as microvascular invasion and data regarding response to pre-LT neoadjuvant therapies are incomplete in the CLTR database and this precludes us from comparing our model with other prominent risk scores. Lastly, although the follow-up time was enough to pick up the events, it is relatively short in comparison with other studies.

## Conclusions

In conclusion, a preoperative predictive risk score for early recurrence of HCC after LT was constructed using 3 variables (pre-LT AFP, largest tumor diameter, and the number of nodules). Our model accurately predicts early recurrence of HCC at an individual level in patients with HBV-cirrhosis. It could effectively classify HCC patients into subgroups with a low, medium and high risk of recurrence and can potentially be used to guide therapeutic decisions and facilitate risk communication between surgeons and patients. We believe that our model can play a complementary role in the selection of HCC patients for LT and prediction of early recurrence alongside the conventional selection criteria. Prospective validation of our model will be an important step to verify its clinical utility.

## Data Availability

The data that support the findings of this study are available from the China Liver Transplant Registry but restrictions apply to the availability of these data, which were used under license for the current study, and so are not publicly available. Data are however available from the authors upon reasonable request and with permission of the China Liver Transplant Registry.

## Abbreviations

AFP: Alpha-fetoprotein
BMI: Body mass index
CI: Confidence interval
CLTR: China Liver Transplant Registry
CVA: Cerebrovascular accident
DBCD: Donation after brain death followed by circulatory death
DBD: Donation after brainstem death
DCD: Donation after circulatory death
HBV: Hepatitis B virus
HCC: Hepatocellular carcinoma
HR: Hazard ratio
IQR: Interquartile range
LT: Liver transplantation
MELD: Model for End-Stage Liver Disease
